# Density and characteristics of tree cavities inside and outside Volcanoes National Park, Rwanda

**DOI:** 10.1002/ece3.9461

**Published:** 2022-10-27

**Authors:** Augustin Niringiyimana, Alphonse Nzarora, Jean Claude Twahirwa, Yntze van der Hoek

**Affiliations:** ^1^ Department of Biology University of Rwanda Huye Rwanda; ^2^ Dian Fossey Gorilla Fund International Musanze Rwanda

**Keywords:** Albertine Rift, excavators, fungal decay, microclimate, nest web, snag density, tree cavities

## Abstract

Tree cavities, formed by animal excavation or processes of fungal decay and mechanical damage, may provide nesting, roosting, or resting opportunities to many invertebrate and vertebrate species. Although cavity availability has been linked to patterns of biodiversity and ecosystem functioning elsewhere, there have been few such studies in the Afrotropics. Here, we present a baseline survey of cavity availability inside the high elevation (2200–3714 m) Afromontane forest ecosystems of Volcanoes National Park (VNP), Rwanda. We aimed to provide such reference data in the form of summary statistics on cavity density and characteristics in a collection of 400 m^2^ plots that together cover 8.8 ha inside and 0.68 ha outside VNP. We also explored the relative importance of fungal decay vs. excavators in the formation of cavities, tested for the relative role of standing dead trees and living trees as cavity substrates, considered differences in diameter and height between cavity‐bearing trees and trees without cavities, tested whether cavity density varies across elevation, and determined the orientation of cavity entrances. We found 109 cavities in 52 cavity‐bearing trees (dominated by *Hagenia abyssinica*) inside VNP, for a density of 12.4 cavities and 5.9 cavity‐bearing trees per hectare, and none outside the park. More cavities were decay‐formed (*n* = 90) than excavated (*n* = 19), and though most cavities were found in living trees (*n* = 44), the number of cavities in dead trees (*n* = 8) was high relative to dead tree substrate availability. We also found that cavity‐bearing trees were larger than those without cavities, that excavated cavities were predominantly oriented toward the southeast and decay‐formed cavities to the northeast, and that cavity density declined with increases in elevation. Our results show that large and dead trees of particular species are important cavity substrates that need to be given attention in conservation and management, as is clearly illustrated by the lack of cavities in the highly managed *Eucalyptus* stands outside VNP.

## Graphical abstract

Tree cavity availability, an important nest and shelter site for a host of species that are linked to patterns of biodiversity and ecosystem functioning elsewhere, has been given little attention to the Afrotropics. A baseline survey of cavity availability inside the high‐elevation (2200–3714 m) Afromontane forest ecosystems of Volcanoes National Park, Rwanda, showed that large and dead trees of particular species are important cavity substrates that need to be given attention in conservation and management.

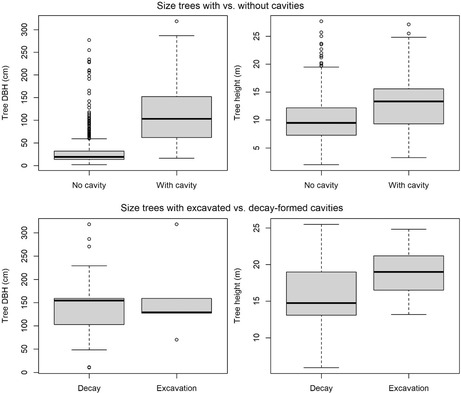

## 1 INTRODUCTION

Tree cavities, either excavated by woodpeckers (Picidae) or other birds or created by mechanical damage and fungal decay, are useful for many animals as substrates for shelter or nests (Cockle, Martin, & Wesołowski, 2011; Cockle, Martin, & Wiebe, 2011; Trzcinski et al., 2021; van der Hoek et al., 2017; van der Hoek, Faida, et al., 2020; van der Hoek, Gaona, et al., 2020). Cavity users may be classified as excavators (e.g., aforementioned woodpeckers) or secondary cavity users (e.g., parrots [Psittaciformes]), the latter depending on existing cavities (Martin & Eadie, 1999). Because of the essential role of cavities for both types of cavity users, followed by associated or cascading ecological interactions, we may find cavity density to be indicative of other elements of forest systems and biodiversities such as richness, intactness, or resilience (Cockle et al., 2012; Hardenbol et al., 2019; Ibarra et al., 2020; Micó et al., 2015). But to effectively gain insights into forest ecosystems from data on cavity density and characteristics, as well as the value of those cavities for cavity users, we first need a region‐specific baseline understanding of cavity availability (Cockle, Martin, & Wesołowski, 2011; Cockle, Martin, & Wiebe, 2011). Unfortunately, we lack insights into processes of cavity formation, availability, and use for most of the Afrotropics (but see Downs & Symes, 2004).

A baseline understanding of cavity availability, itself determined by the rate at which cavities are formed or destroyed in a given habitat (Edworthy et al., 2012), aids our efforts to study forest communities and their interactions via “nest webs” (Martin & Eadie, 1999), the survival of the cavity users/nesters (Cornelius et al., 2008), or broader aspects of ecosystem functioning (Ibarra et al., 2020). Multiple biotic and abiotic factors drive spatial variation in tree cavity availability. First, tree cavity availability has been linked to woodpecker abundance and diversity (Styring & Zakaria, 2004), though the evidence is skewed toward temperate ecosystems of the northern hemisphere, and excavator richness itself may be indicative of the diversity of the wider forest bird community (Drever et al., 2008; van der Hoek, Faida, et al., 2020; van der Hoek, Gaona, et al., 2020). Second, tree cavity density may vary across landscapes because of variations in tree species and characteristics (Schepps et al., 1999; Zheng et al., 2018). For example, excavators may disproportionally select large dead trees as nest cavity substrates in some regions (Cockle, Martin, & Wesołowski, 2011; Cockle, Martin, & Wiebe, 2011); though an opposite pattern was found in at least one study in North Africa (Touihri et al., 2015). As a result, cavity density may be representative of the relative availability of large dead trees, which by itself is an indication of the degree of human disturbance and the intactness of forest structure (Wirth et al., 2009). Third, cavity densities vary spatially following patterns of precipitation—a determinant of fungal decay (Remm & Lõhmus, 2011; Zheng et al., 2018). And finally, forest management and human activities such as logging and habitat conversion in general, tend to alter the availability of specific trees (e.g., dead standing trees known as snags) that serve as suitable substrates for nest sites for many species (Cornelius et al., 2008; Politi et al., 2009).

The overall lack of data on tree cavities across the Afrotropics also applies to Volcanoes National Park (VNP), Rwanda. This protected area harbors upper montane mixed forests and is surrounded by agricultural land with no native forest vegetation and few stands of trees dominated by *Eucalyptus* sp. (Akinyemi, 2017). To provide a baseline for future studies on nest‐web interactions and their links with forest functioning, we determined cavity availability and characteristics both inside VNP and in *Eucalyptus* stands outside the park.

We provide several descriptive statistics on the availability of cavities by origin (decay‐formed vs. excavated), location (trunk vs. branch, height on tree), and entrance orientation; the latter being of importance in processes of wood decay and thermoregulation of nest cavities (Rendell et al., 1994). Cavity entrance orientation has been shown to deviate from random in many regions, likely following climatic patterns that govern internal cavity conditions (Landler et al., 2014). Next, we summarized the characteristics of the cavity substrates such as the health (dead vs. living), size (diameter and height), and species of substrate trees, before moving on to a set of predictions. Like cavity characteristics themselves, the characteristics of substrates are determined by various characteristics of nest webs (e.g., cavity user and tree community composition) and the range of abiotic conditions discussed above (see syntheses by Cockle, Martin, & Wesołowski, 2011; Remm & Lõhmus, 2011). First, as our study area sees high amounts of precipitation, we predicted that fungal decay would be a key agent in cavity formation (Boyle et al., 2008). Second, given findings on cavity availability and excavator preference elsewhere, we also predicted that cavity‐bearing trees would be larger in both diameter and height than trees without cavities (Cockle, Martin, & Wesołowski, 2011; Cockle, Martin, & Wiebe, 2011; Edworthy et al., 2017). Larger and older trees tend to have experienced long periods of decay and are more attractive to excavators due to their capacity to support cavities with relatively large dimensions (Lindenmayer et al., 2000; Rudolph & Conner, 2016). Finally, we predicted that cavity density would decrease with an increase in elevation due to a decline in excavator abundances and suitable cavity substrates at higher elevations near the treeline ecotone (Altamirano et al., 2015).

## 2 METHODS

### 2.1 Study area

**FIGURE 1:**
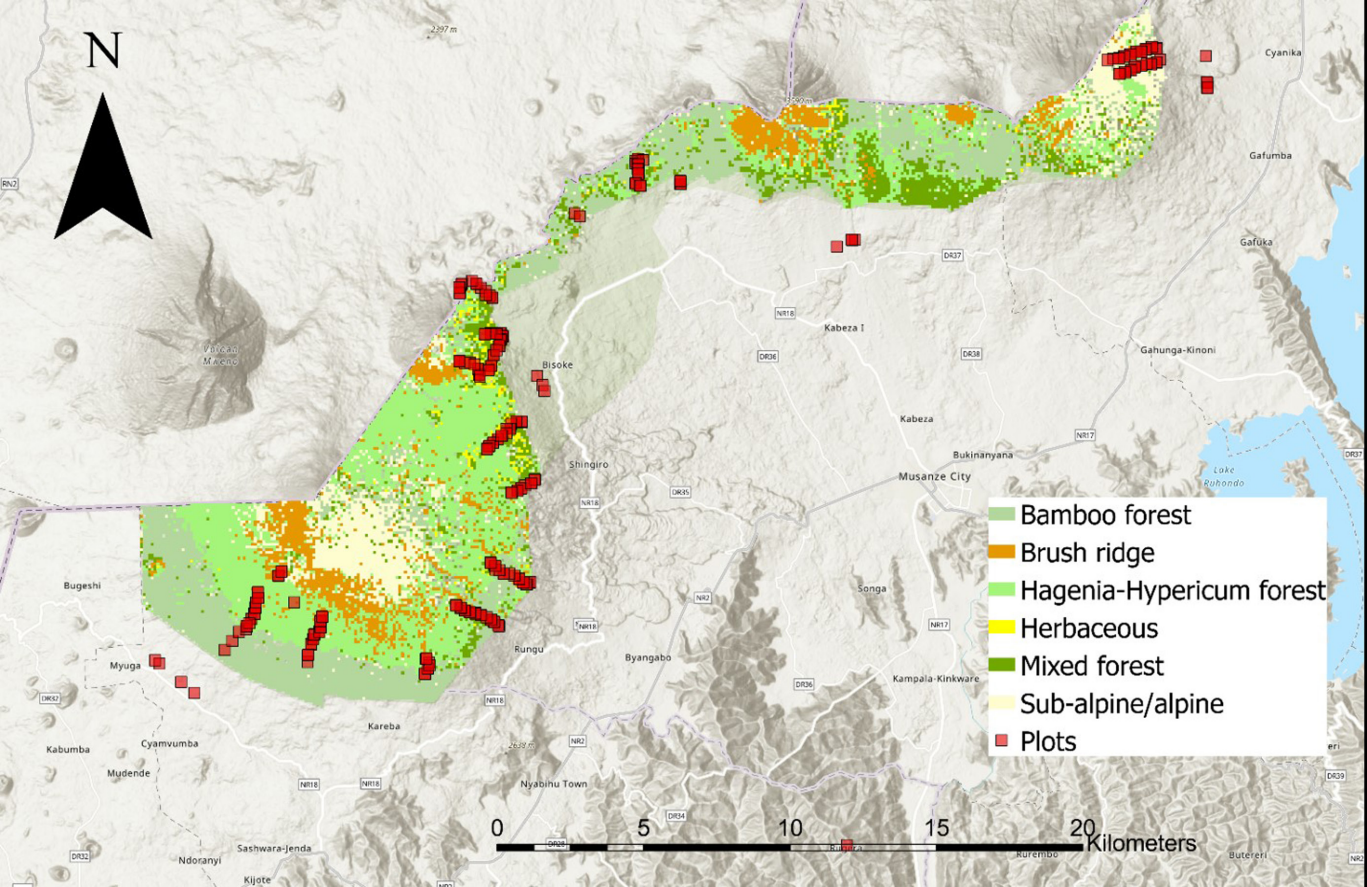
Location of plots used to sample tree cavity density inside and outside Volcanoes National Park, Rwanda. Plots outside the borders of Volcanoes National Park (colored area) were located in *Eucalyptus* stands.

We surveyed cavity availability in plots inside and up to 1 km outside Volcanoes National Park, in northern Rwanda (~1°30′S, 29°30′E; Figure 1). Plots covered an elevational range of ~2200–3700 m a.s.l. and various vegetation types: *Eucalyptus* stands outside VNP at 2200–2638 m, mixed montane forest at 2500–2700 m; bamboo (*Yushania alpina*) forest at 2500–2800 m; *Hagenia ‐ Hypericum* forest at 2800–3300 m; and *Hypericum* woodlands (“brush ridge”) at 3000–3700 m (Akayezu et al., 2019).

There are limited climatic data available for VNP from field stations, but estimates based on 30 arc‐second resolution data (CHELSA) indicate a mean annual air temperature of 14.5°C and mean daily maximum air temperature of the warmest month of 20.2°C at 2300 m a.s.l., the park's lower elevation boundaries (Karger et al., 2017). These temperatures drop to 8.4 and 12.5°C, respectively, at the peak of the volcanoes (~4300 m a.s.l.). Annual precipitation peaks in two wet seasons (March–May, September–December), and annual averages are between 1570 and 1700 mm.

The cavity‐using avifauna of VNP includes at least three species of excavator (olive woodpecker *Chloropicus griseocephalus*, western tinkerbird *Pogoniulus coryphaea*, yellow‐rumped tinkerbird *Pogoniulus bilineatus*) and four secondary cavity nesters (spotted eagle‐owl *Bubo africanus*, stripe‐breasted tit *Melaniparus fasciiventer*, African wood‐owl *Strix woodfordii*, barn swallow *Hirundo rustica*), though our understanding of the breeding behavior of most African birds is notably limited and additional species may be facultative cavity users (van der Hoek et al., 2017). In addition to birds, several other animals utilize cavities, such as the southern tree hyrax (*Dendrohyrax arboreus*) and bees (Apoidea).

### 2.2 Tree cavity sampling methods

Between June and August 2019, we recorded cavities in 220 plots inside and 17 plots outside VNP, allocated to encompass a range of elevations and a representative—with regards to the area covered by respective vegetation types—sampling of dominant vegetation types (17 plots in *Eucalyptus* stands, 46 in bamboo, 72 in mixed forest, and 102 in *Hagenia ‐ Hypericum* forest and *Hypericum* woodlands; Figure 1). Each plot measured 20 × 20 m, for a total surface area covered of 8.8 ha inside and 0.68 ha outside VNP. We opted for many relatively small (400 m^2^) plots over few larger (e.g., the 0.5 ha plots used by Boyle et al., 2008) plots as (i) we aimed to sample across multiple vegetation types and elevations and were (ii) hindered by the steep terrain in our efforts to establish precisely measured large plots. Plots, the locations of which were governed by our efforts to link data collected for other research (e.g., van der Hoek et al., 2021) and logistic constraints, were laid out along transects that followed the elevational gradient. Given that each of these small plots will only include a few trees, we were wary of under‐ or overestimation of cavity densities and thus aimed to increase the surface area covered at each site by establishing pairs of plots, each separated by 10 m, along the transects. There were a few exceptions to this approach, in cases where there were zero trees at the location of the paired plot (e.g., if there was only agricultural land next to a small patch of *Eucalyptus*), and we effectively sampled cavity availability at 126 distinct geographical locations. Each pair of plots was separated by 200 m from the nearest pair of plots and transects were at least 1000 m apart.

In each plot, we measured the height and Diameter Breast Height (DBH) of all trees >1 m high and >10 cm DBH. We checked each tree for the presence of cavities, using binoculars to scan the upper part of the higher trees. Cavities were solely checked from the ground, though efforts were made to determine the approximate depth of cavities, for example by zooming in on photographs. We recorded those cavities, which we deemed suitable for use, in that a bird would be able to enter entirely (minimum ~3 cm entrance diameter), and omitted smaller depressions or cavities that were too small for a bird to be sheltered from the outside. For each tree, we recorded the species, the presence of cavities, and the health status of the tree (living vs. dead). For cavity‐bearing trees, we also recorded the most likely agent of formation (excavated vs. decay‐formed) of each cavity, the orientation of the cavity entrances (in degree), and location of the cavities (trunk vs. branch), and the height of the cavities on the tree. We considered cavities most likely to be excavated if their entrances were regularly circular or oval‐rectangular in shape whereas irregularly shaped cavities were deemed decay‐formed.

### 2.3 Statistical analysis

We first determined the densities of excavated and decay‐formed cavities, as well as that of cavity‐bearing trees, inside and outside VNP. For this, we converted densities from the 400 m^2^ plot surface to per hectare densities. We also specified how many of these cavity‐bearing trees could be classified as dead or living trees. We subsequently used a chi‐square test to compare the relative number of dead and living trees with cavities.

Next, we fitted a series of nonlinear Generalized Additive Models (GAMs) to the relationships between the predictor variable elevation and various response variables related to cavity or substrate availability and characteristics. For this, we summed the data from the paired plots, retaining one data point per geographical location (i.e., reducing the 237 plots to 126 samples, see Tree cavity sampling methods). We considered the response variables cavity, tree, and snag densities to follow a zero‐inflated Poisson distribution, whereas models for response variables tree DBH and height were fitted according to a Gaussian distribution. We fitted a separate model for each response variable. As cavity density itself may be dependent on tree (substrate) density, we also used AIC model selection to compare the simple model for cavity density with a model that included tree density as an offset. We fitted GAMs using the “mgcv” package in R (Wood & Wood, 2015).

Last, we focused on the characteristics of the cavity‐bearing trees and cavities. First, we used Wilcoxon signed‐rank tests to compare the DBH and height of cavity‐bearing trees with trees without cavities. Next, we determined the location of cavities as being in tree branches or trunks, estimated the mean heights of cavities on trees, and used Wilcoxon signed‐rank tests to compare the height and DBH of trees with excavated and decay‐formed cavities. Similarly, we analyzed the orientation (aspect in degrees) of cavity entrances and compared these between cavity‐forming agents. For the latter, we performed Rao's spacing test (“circular” package in R [Agostinelli, 2007]) to evaluate whether the distribution of aspects of excavated and decay‐formed cavities deviated significantly from random, showing some uniform directionality, and calculated the associated mean directions of aspects plus the circular standard deviation (Landler et al., 2014). We used the Mardia–Watson–Wheeler test, available in the same circular package, to test for differences in the orientation of excavated and decay‐formed cavities.

## 3 RESULTS

We found 109 cavities in 52 cavity‐bearing trees inside VNP, for a density of 12.4 cavities and 5.9 cavity‐bearing trees per hectare, and none in the *Eucalyptus* stands surveyed outside the park (Table 1)—relative to an average tree density of 134.7 per hectare across plots inside VNP; plots outside were selected on their high tree cover, which is not representative of the large open agricultural landscape. This implied that inside VNP, approximately 4% of trees contained at least one cavity. Considering data from the park only, we found that a mere 19 cavities (17% of cavities) were excavated by birds (15 cavities in six living trees, four in two dead trees), for a density of 2.2 excavated cavities and 0.9 excavated‐cavity‐bearing trees per hectare. Of the 90 remaining decay‐formed cavities (83% of cavities), 82 were located in 43 living trees and eight in eight dead trees, for a density of 10.2 decay‐formed cavities and 5.8 decay‐formed‐cavity‐bearing trees per hectare. We note that some trees contained multiple cavities, occasionally even a mix of cavities of decay‐formed and excavated origin. Following these overall higher numbers of cavities in living versus dead trees, we found that the relative number of dead trees with cavities was significantly different from that of living trees with cavities (*χ* ^2^ = 24.923, df = 1, *p* < .001).

**TABLE 1:** Density of cavity‐bearing trees per hectare inside and outside Volcanoes National Park, Rwanda, and the likely causal agent of cavity formation.

Cavity	No	Yes		Total
Agent	NA	Bird excavated	Decay	
Inside	128.8	0.9	5.8	134.7
Dead	13.4	0.2	0.9	14.3
Living	115.3	0.7	4.9	120.3
Outside	161.8	0	0	161.8
Dead	1.5	0	0	1.5
Living	160.3	0	0	160.3
Total	131.1	0.8	5.4	136.6

*Note*: NA refers to trees without cavities. Note that some trees contained both excavated and decay‐formed cavities.

**FIGURE 2:**
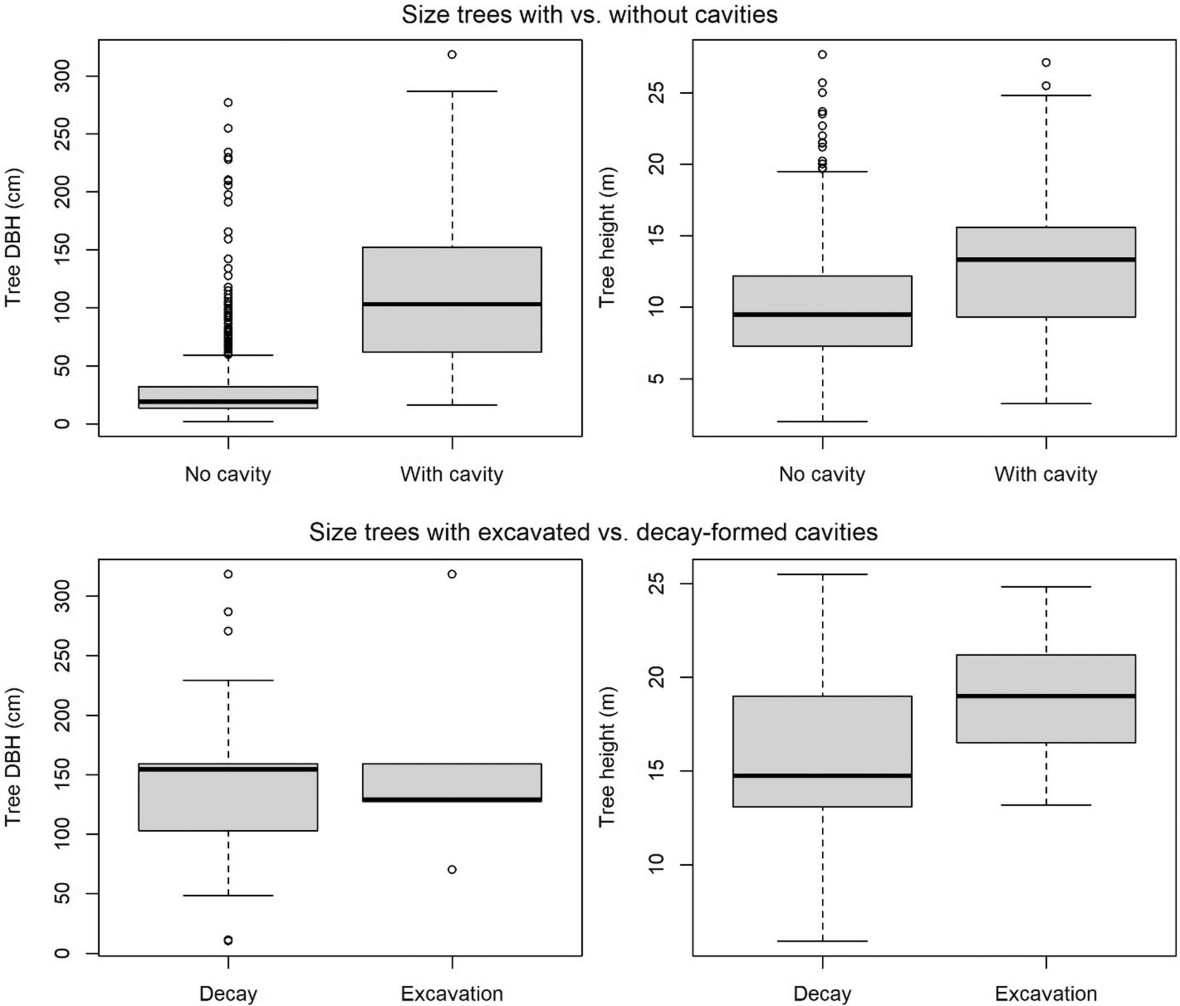
Boxplots of the range of sizes (diameter breast height [DBH] and height) of trees with (*n* = 52) and without cavities (*n* = 1133), with the former further specified as trees with excavated cavities (*n* = 8) and decay‐formed cavities (*n* = 51), inside Volcanoes National Park (Rwanda). The whiskers represent the upper and lower quartiles, and the vertical black line indicates the median. Note that some trees had both excavated and decay‐formed cavities.

**FIGURE 3:**
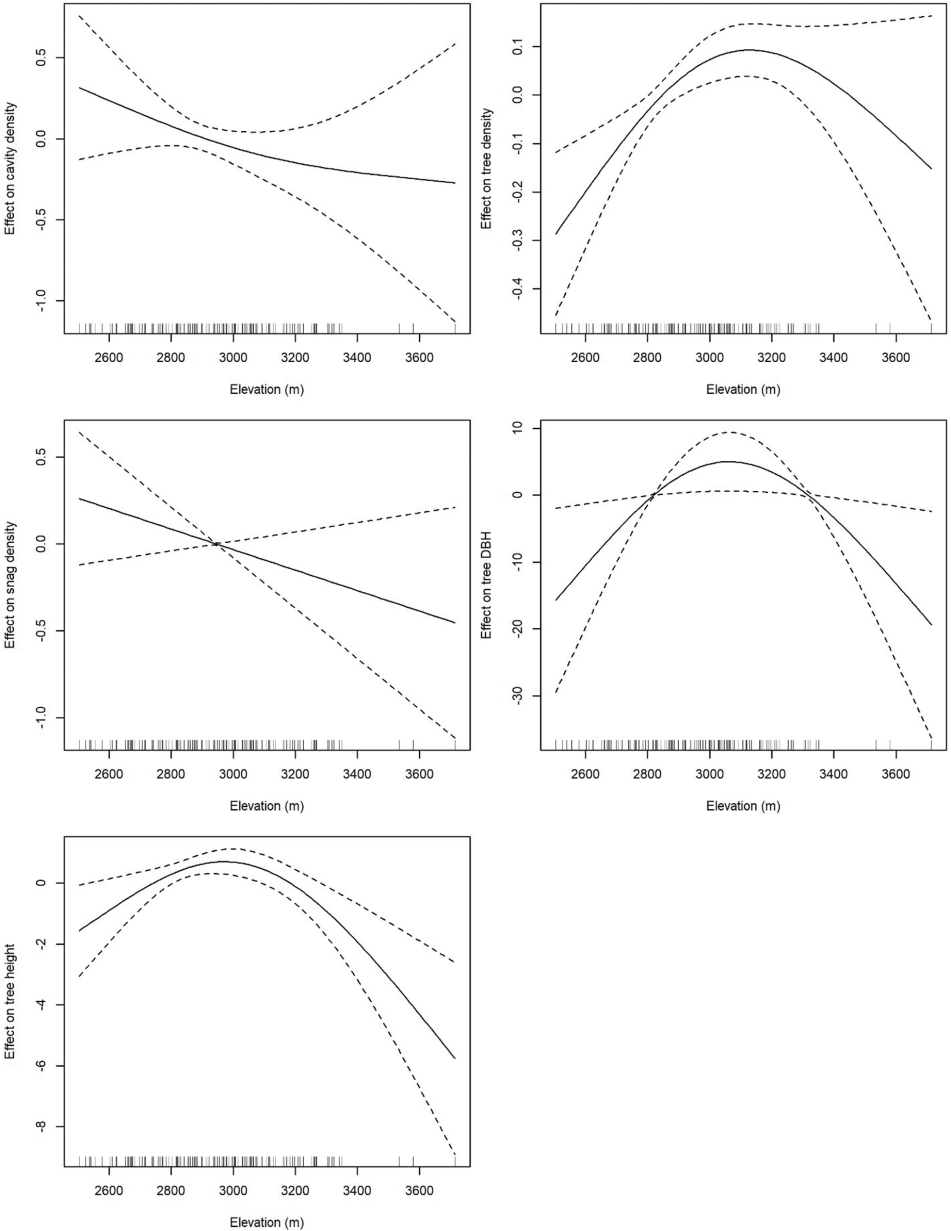
Effect of plots of smooth functions of generalized additive models (GAMs) fit to elevation (predictor) and cavity density, tree density, snag density, mean tree DBH, and mean tree height (response variables). The full lines represent the positive or negative effect of elevation on any of the response variables with the dashed lines the upper and lower twice‐standard error curves of the predicted effect.

*Hagenia abyssinica* harbored the highest absolute (10 excavated, 48 decay‐formed cavities) and relative (25.0% of trees of this species contained at least one cavity) number of cavities, in absolute numbers followed by *Hypericum revolutum* (two excavated, 24 decay‐formed cavities; 1.7% of trees of this species) and *Dombeya goetzenii* (six excavated, seven decay‐formed cavities; 20.0% of trees of this species; Table 2)—several trees contained multiple cavities thus the number of cavity‐bearing trees was lower than that of cavities. We also found that cavity‐bearing trees were significantly larger than trees without cavities, in both DBH (median with cavity 103.2 cm, SD = 70.7; median without cavity 19.1 cm, SD = 28.8; *W* = 4679, *p* < .001) and height (median with cavity 13.3 m, SD = 5.5; median without cavity 9.5 m, SD = 3.7; *W* = 19,320, *p* < .001; Figure 2). We found that cavity density declined with increasing elevation, with a GAM showing a near‐significant effect of elevation on absolute cavity density (edf = 0.821, Ref.df = 2, *χ* ^2^ = 2.216, *p* = .085; Figure 3). Once corrected for variation in tree density (included as an offset in the GAM), this effect became significant (edf = 1.995, Ref.df = 2, *χ* ^2^ = 703.7, *p* < .001), though we caution that this model had a much lower fit than the simple model (∆AIC = 1158.1). The density of dead trees followed a similar decline with elevation, though this effect was not significant (edf < 0.646, Ref.df = 2, *χ* ^2^ = 1.882, *p* = .088), while tree density (edf = 1.505, Ref.df = 2, *χ* ^2^ = 11.810, *p* < .001), DBH (edf = 0.843, Ref.df = 2, *χ* ^2^ = 2.614, *p* = .014), and height (edf = 1.789, Ref.df = 2, *χ* ^2^ = 7.133, *p* < .001) all peaked at ~2800–3200 m (Figure 3).

**TABLE 2:** Counts and percentage (in parentheses) of trees (>1 m high and >10 cm DBH) with and without cavities in selected plots representing ~9.5 hectare of Volcanoes National Park (Rwanda) and surrounding landscape.

Species	Dead		Living		All	
	No cavity	With cavity	No cavity	With cavity	No cavity	With cavity
*Hagenia abyssinica*	7	4 (36.4)	71	22 (23.7)	78	26 (25.0)
*Hypericum revolutum*	86	2 (2.3)	603	10 (1.6)	689	12 (1.7)
*Dombeya goetzenii*	4	1 (20.0)	20	5 (20.0)	24	6 (20.0)
*Faurea saligna*	0	1 (100.0)	57	3 (5.0)	57	4 (6.6)
*Cornus volkensii*	2	0	14	3 (17.6)	16	3 (15.8)
*Maesa lanceolata*	4	0	23	1 (4.2)	27	1 (3.6)
*Eucalyptus maidenii*	1	0	99	0	100	0
*Xymalos monospora*	1	0	85	0	86	0
*Prunus africana*	3	0	50	0	53	0
*Philippia johnstonii*	3	0	36	0	39	0
*Psychotria mahonii*	2	0	32	0	34	0
*Alnus acuminata*	0	0	12	0	12	0
*Cupressus* sp.	1	0	10	0	11	0
*Ilex mitis*	0	0	5	0	5	0
*Acacia melanoxylon*	0	0	4	0	4	0
*Bersama abyssinica*	0	0	2	0	2	0
*Galiniera coffeoides*	1	0	2	0	3	0
*Ficus thonningii*	0	0	1	0	1	0
*Neoboutonia macrocalyx*	0	0	1	0	1	0
Unknown	4	0	0	0	4	0

*Note*: We calculated the percentage of cavity‐bearing trees, per health status (dead or living) or across all individuals, for each tree species separately.

**FIGURE 4:**
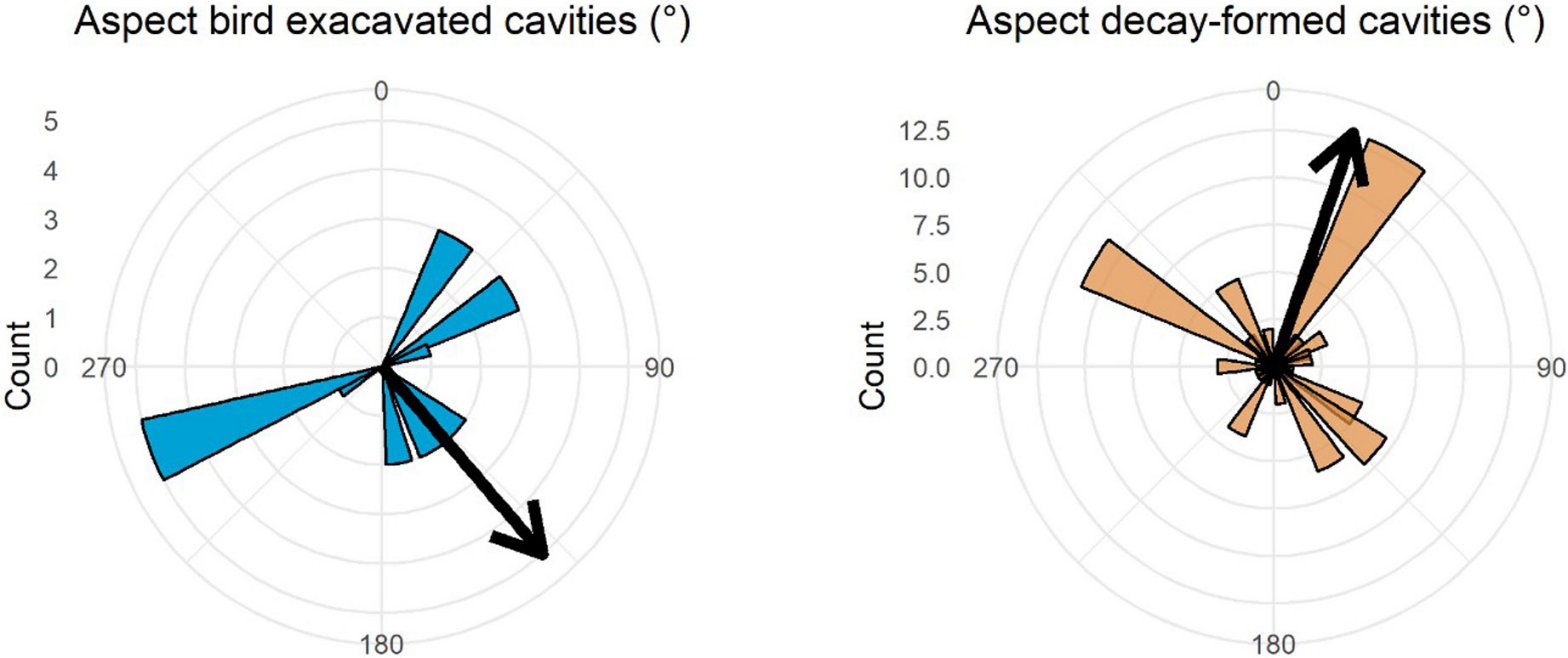
Circular distribution of aspects of entrances of excavated (*n* = 19) and decay‐formed cavities (*n* = 90) in Volcanoes National Park, Rwanda. Colored bars represent the cavity counts in each 15‐degree bin, and the arrows represent the mean aspect.

With regards to the location of cavities on trees, we found 12 excavated cavities in tree branches and seven in tree trunks, whereas 31 decay‐formed cavities were located in branches and 59 in tree trunks. Although excavated cavities were located significantly higher above the ground (median 6.70 m, SD = 1.50) than decay‐formed cavities (median 2.35 m, SD = 4.11; *W* = 521.5, *p* = .008), we found no significant difference in the size of trees with excavated (median DBH = 129.0 cm, SD = 69.2; median height = 19.0 m, SD = 3.6) versus those with decay‐formed cavities (median DBH = 154.5 cm, SD = 14.7; median height = 14.7 m, SD = 4.6; DBH: *W* = 253, *p* = .794; height: *W* = 152, *p* = .069; Figure 2). Finally, with regards to the orientation of cavities, we found that the entrances of both excavated (mean aspect 139.5°, SD = 1.8) and decay‐formed cavities (mean aspect 18.6°, SD = 1.7) showed a significant departure from a random distribution (Rao's *U* = 222.30, *p* < .001 for excavated, Rao's *U* = 249.76, *p* < .001 for decay‐formed cavities; Figure 4). Despite the differences in mean aspects, we found no evidence of a significant difference in the orientation of cavities formed by excavation or decay (Mardia–Watson–Wheeler test, *W* = 4.44, *p* = .109).

## 4 DISCUSSION

Densities of cavities and cavity‐bearing trees inside Volcanoes National Park were low compared with some other tropical regions (e.g., Thailand: 189–407 cavities/ha, Pattanavibool & Edge, 1996; Costa Rica: 112 cavities/ha, Boyle et al., 2008; Mexico: 37 cavities/ha, Vázquez & Renton, 2015), but fall with the overall broad range of densities found in the tropics (as summarized by Remm & Lõhmus, 2011). There was a complete lack of cavities in the *Eucalyptus* stands that neighbor the park. Higher numbers of decay‐formed than excavated cavities suggested that fungal decay is a particularly important cavity‐forming agent in VNP, with dead and large trees—particularly *H. abyssinica*—being the most important cavity substrates, a pattern which reflects that of several primary forests in other geographic regions (Cockle et al., 2010; Wesołowski, 2007). Cavity densities decline with elevation while tree densities and sizes peak around ~3000–3100 m. Decay‐formed and excavated cavities varied in location on trees and characteristics, with decay‐formed cavities most often located in tree trunks and oriented to the northeast, and excavated cavities mainly located in branches and facing southeast.

That cavity densities in and outside VNP are rather low as compared to some other tropical regions may be related to past and current human intervention in the region, such as tree cutting or tree harvesting (Munanura et al., 2018). The lack of cavities outside VNP is likely to be linked to the replacement of native vegetation with highly managed *Eucalyptus* stands (Akinyemi, 2017), which has reduced the availability of large trees in advanced stages of decay as suitable substrates for cavity formation. We found no dead trees in the plots located outside the park. In addition, the *Eucalyptus* stands found outside VNP are near‐complete monocultures, with no other potential cavity substrates mixed in, unlike *Eucalyptus* plantations in, for example, Tanzania where the presence of the occasional *Macaranga capensis* tree provides a substrate for cavity nesters (John & Kabigumila, 2007). Relatively low cavity densities inside VNP may be related to the low density of trees at these high elevations, and subsequently that of trees of the size required to contain cavities that are potentially suitable as nest sites (e.g., ≥60 cm in subtropical South America; Cockle et al., 2010). Indeed, our estimate of 134.7 trees per hectare is well below the interquartile range of tree densities for either tropical moist or dry forest (lower quartile cut‐off >250 trees per hectare for either forest type, Crowther et al., 2015). In addition, but related to low tree availability at high elevations near the treeline ecotone, there is a potentially low abundance and richness of excavators (avifauna) in VNP (van der Hoek et al., 2021; van der Hoek, Faida, et al., 2020; van der Hoek, Gaona, et al., 2020).

Decay‐formed and excavated cavities were found on different parts of trees (trunk vs. branch) and were oriented in different directions, which may be related to solar radiation and precipitation. These climatic factors are known to influence the hardness of woody tissue, rates of wood decay, and internal cavity microclimate conditions (Ojeda et al., 2021). The particularly high prevalence of excavated cavities in branches may be related to excavators' preferences for softer wood for excavation of cavities (Schepps et al., 1999), though follow‐up studies would need to confirm that branches differ from trunks in wood tissue hardness. With regards to orientation, we find that most trees in our study area grow on slopes that face a southern or eastern exposure, and high decay‐inducing levels of precipitation and moisture may be reached on the northwestern slope‐facing sides of trees as precipitation increases with elevation (van der Hoek et al., 2021). Following this, we may also assume that excavated cavities are predominantly found on the northwestern side of trees where the relatively softer wood would be easier to excavate. Instead, we found a predominantly southeastern orientation of excavated cavities, in line with that found near the equator in South America (van der Hoek, 2017). Tentatively, this preference could be related to the maintenance of a microclimate in cavities favorable for reproductive success (Inouye et al., 1981; Paclík & Weidinger, 2007) though it is uncertain which temperatures or levels of humidity would constitute an optimal microclimate. For example, a study in temperate forests showed that cavities, under natural conditions, may experience humidity levels as high as 90% saturation (Maziarz et al., 2017).

Maintaining both the availability of tree cavity substrates and cavity‐forming processes is important for the conservation of functional and diverse forests. For VNP and immediate surroundings, this requires a focus on the retention of large trees, preferably of native species such as *H. abyssinica* and *D. goetzenii*, in advanced stages of decay (Schaaf et al., 2020). We recommend that future studies build on the baseline data provided in this study, particularly by addressing cavity use and nest‐web interactions, to better understand the role of these key habitat elements in the Afromontane forests of Central and East Africa.

## AUTHOR CONTRIBUTIONS

**Augustin Niringiyimana:** Data curation (lead); formal analysis (lead); software (equal); writing – original draft (equal); writing – review and editing (equal). **Alphonse Nzarora:** Formal analysis (equal); project administration (lead); supervision (equal); visualization (equal); writing – review and editing (equal). **Jean Claude Twahirwa:** Data curation (equal); visualization (equal); writing – review and editing (equal). **Yntze van der Hoek:** Conceptualization (lead); formal analysis (equal); methodology (equal); project administration (equal); supervision (lead); writing – original draft (equal).

## CONFLICT OF INTEREST

None declared.

## Data Availability

All raw georeferenced data, summarized by plots, can be found in Supporting Information on Dryad: https://doi.org/10.5061/dryad.2jm63xssd.
